# Supplementary material for: HBV vaccination and PMTCT as elimination tools in the presence of HIV: insights from a clinical cohort and dynamic model
Source: BMC Med. 2019 Feb 21;17:43. doi: 10.1186/s12916-019-1269-x (PMC6383254; doi:10.1186/s12916-019-1269-x)
Supplement: Supplementary file 1 — Clinical cohort metadata. Metadata for three paediatric cohorts recruited in Kimberley, South Africa, including longitudinal CD4+ T cell and viral load data for paediatric HIV cohort age ≤ 60 months in Kimberley, South Africa. HBV vaccine responses.csv. HBV vaccine responses.xlsx. COSAC cohort Tables 1 and 2.docx. These files are available on-line using the following link: https://doi.org/10.6084/m9.figshare.5601679. (ZIP 239 kb) [file 12916_2019_1269_MOESM1_ESM.zip › COSAC cohort tables 1 and 2.docx]

**The impact of hepatitis B virus (HBV) vaccination on**

**infection and immunity in South African children**

**DATA TABLES**

**Table 1: Characteristics of three paediatric study cohorts, comprising 402 children, recruited from Kimberley Hospital, South Africa.**

| **Cohort** | **HIV negative;**  **KReC**  **(age ≤60 months)** | **HIV positive**  **(age ≤60 months)** | **HIV positive**  **(age >60 months)** |
| --- | --- | --- | --- |
| **Number of subjects** | 174 | 136 | 92 |
| **Age range in months** | 8-58 | 6-60 | 64-193 |
| **Median age in months (IQR)** | 18 (12-26) | 29 (18-40) | 137 (122-154) |
| **Sex (% male)** | 55.4 | 44.9 | 45.6 |

KReC = Kimberley Respiratory Cohort. IQR = interquartile range.

Table 2: Detailed information and serological profiles of five children from Kimberley, South Africa, with serological evidence of current or previous infection with HBV (based on positive HBsAg (n=3) or anti-HBc (n=2))

| **Subject ID** | **K306** | **K405** | **KReC51** | **KReC151** | **K093** |
| --- | --- | --- | --- | --- | --- |
| **Cohort** | HIV+  age ≤60 months | HIV+  age ≤60 months | KReC | KReC | HIV+  age >60 months |
| **Sex** | F | F | F | M | F |
| **Age (months) at time of sampling** | 18 | 37 | 20 | 15 | 118 |
| **HIV infection** | Positive | Positive | Negative | Negative | Positive |
| **ART^a^ (if HIV positive)** | Yes | Yes | n/a | n/a | No |
| **Number of doses of HBV vaccine** | NK | NK | NK | 3 | NK |
| **HBsAg result^b^** | Detected | Detected | Detected | Not detected | Not detected |
| **Anti-HBc result^c^** | Not detected | Not detected | Detected | Detected | Detected |
| **HBeAg result^d^** | Not done | Not done | Detected | Not done | Not done |
| **Anti-HBs result^e^** | Not detected | Not detected | Not detected | Detected | Not detected |
| **Interpretation** | Active infection | Active infection | Active infection | Immunised, infected and cleared | Infected and cleared |

^a^ART indicates the participant was receiving anti-retroviral therapy to treat HIV infection; ^b^Hepatitis B surface antigen test; ^c^Hepatitis B core antibody test; ^d^Hepatitis B envelope antigen test; ^e^Hepatitis B surface antibody test (vaccine mediated response). KReC = Kimberley Respiratory Cohort. n/a = not applicable.
