# Supplementary material for: HBV vaccination and PMTCT as elimination tools in the presence of HIV: insights from a clinical cohort and dynamic model
Source: BMC Med. 2019 Feb 21;17:43. doi: 10.1186/s12916-019-1269-x (PMC6383254; doi:10.1186/s12916-019-1269-x)
Supplement: Supplementary file 2 — Full model description and additional supplementary Figure S1-S6. This document includes a complete model description, including parameters, assumptions, mathematical expressions and fitting details, including further results and figures supporting the main text: Figure S1. Posteriors from the Bayesian Markov Chain Monte Carlo fitting to the Kimberley cohort data. Figure S2. Fitting HBV vaccine response according to HIV serostatus. Figure S3. Sensitivity of interventions with deterministic output. Figure S4. Post-intervention stochastic impact on HBV prevalence (HBsAg), with time to reach sustainable development goals when using routine neonatal vaccination and PMTCT independently. Figure S5. Sensitivity of mean intervention impact on HBV incidence (HBsAg) and HBeAg+ prevalence, with estimated mean year to reach sustainable development goals for combinations of routine + 6 years vaccination and PMTCT. Figure S6. Sensitivity of mean intervention impact on HBV incidence (HBsAg) and HBeAg+ prevalence, with estimated mean year to reach sustainable development goals for combinations of routine neonatal vaccination and PMTCT plus a complete catch-up campaign. (PDF 3606 kb) [file 12916_2019_1269_MOESM2_ESM.pdf]

HBV vaccination and PMTCT as elimination tools in the presence of HIV: insights from a clinical cohort and dynamic model

Additional File 2: Full model description and additional supplementary figures S1-S6

---

*Anna L McNaughton, anna.mcnaughton@ndm.ox.ac.uk

*José Lourenço, jose.lourenco@zoo.ox.ac.uk

*Louise Hattingh, loeise@gmail.com

Emily Adland, emily.adland@paediatrics.ox.ac.uk

Samantha Daniels, danies.samantha@gmail.com

Anriette Van Zyl, ajmvanzyl@yahoo.com

Connie S Akiror, connieakiror@gmail.com

Susan Wareing, susan.wareing@ouh.nhs.uk

Katie Jeffery, katie.jeffery@ouh.nhs.uk

M Azim Ansari, ansari.azim@gmail.com

Paul Klenerman, paul.klenerman@ndm.ox.ac.uk

Philip J R Goulder, philip.goulder@paediatrics.ox.ac.uk

Sunetra Gupta, sunetra.gupta@zoo.ox.ac.uk

Pieter Jooste, jpjooste@mweb.co.za

§Philippa C Matthews, philippa.matthews@ndm.ox.ac.uk

* These authors contributed equally to this work

§ Corresponding author Philippa Matthews

CONTENTS

Full description of model and fitting approaches 3

Framework 3

Carriage and infection types 4

Force of Infection 5

Births and Mortality 5

Vertical Transmission 5

Routine vaccination 6

Catch-up vaccination 6

Bayesian markov-chain Monte-Carlo fitting approach 6

MCMC and model implementation 7

Fitting demographic background 7

Fitting transmission background 7

Fitted parameters and priors for transmission setting 8

Simulating deterministic interventions 8

Simulating stochastic interventions 8

Fitting of cohort data on HIV serostatus and HBV vaccine-induced protection 9

Modelled HBV vaccine-induced protection in the context of HIV status 9

Obtained posteriors for informed and uninformed parameters 10

ADDITIONAL SUPPLEMENTARY figures (Fig S1-S6) 11

Additional Supplementary Figure 1 (Fig S1): Posteriors from the Bayesian Markov Chain Monte-carlo fitting to the Kimberley cohort data. 11

Additional Supplementary Figure 2 (Fig S2): Fitting HBV vaccine response according to HIV serostatus 12

Additional Supplementary Figure 3 (Fig S3): Sensitivity of interventions with deterministic output. 13

Additional Supplementary Figure 4 (Fig S4): Post-intervention stochastic impact on HBV prevalence (HBsAg), with time to reach sustainable development goals when using routine neonatal vaccination and PMTCT independently. 14

Additional Supplementary Figure 5 (Fig S5): Sensitivity of mean intervention impact on HBV incidence (HBsAg) and HBeAg+ prevalence, with estimated mean year to reach sustainable development goals for combinations of routine +6 years vaccination and PMTCT. 15

Additional Supplementary Fig 6 (Fig S6): Sensitivity of mean intervention impact on HBV incidence (HBsAg) and HBeAg+ prevalence, with estimated mean year to reach sustainable development goals for combinations of routine neonatal vaccination and PMTCT plus a complete catch-up campaign 16

References 17

FULL DESCRIPTION OF MODEL AND FITTING APPROACHES

Framework

This model takes into consideration the proportion of the population susceptible to hepatitis B virus (HBV) infection (S), chronic (C) and acute (I) HBV carriers, the immune (R) and the vaccinated (V) (main article, Fig1A). We divided susceptible (S) and vaccinated (V) individuals into three subgroups representing infants (*i*, <1 years of age), children (*c*, 1-6 years of age) and older individuals (comprising older children, adolescents and adults, *a*, >6 years of age). We divided chronic carriers (C) into HBeAg-positive (C+) and HBeAg-negative (C-) to further allow for different parameterization between these two biologically distinct states.

Natural decay of vaccine-mediated immunity and the effects of HIV sero-status on vaccine-induced protection are also taken into account. We used a Bayesian Markov-chain Monte Carlo (bMCMC) approach to fit the dynamic model to the local demographic and epidemiological setting of Kimberley before projecting the impact of interventions. We used informative priors for model parameters for which robust literature support exists, and uninformative (uniform) priors otherwise. For full details on the model and fitting approach, see the Methods section.

The following set of ordinary differential equations (ODE) is used to model the deterministic transmission of HBV under homogeneous mixing. Constant parameters informed by the literature and estimated parameters are described in further detail below.

$$
\begin{aligned}
\frac{d_{S_i}}{d_t} &= Z - cS_i - \lambda S_i - \mu S_i & (1)\\
\frac{d_{S_c}}{d_t} &= (1-\omega_c)cS_i - aS_c - \lambda S_c - \mu S_c & (2)\\
\frac{d_{S_a}}{d_t} &= (1-\omega_a)aS_c - \lambda S_a - \mu S_a & (3)\\
\frac{d_I}{d_t} &= \lambda\gamma S_a + \lambda\epsilon S_c + \lambda\psi S_i & \\
&\quad +\lambda\gamma(1-\Delta_a)V_a + \lambda\epsilon(1-\Delta_c)V_c + \lambda\psi(1-\Delta_i)V_i & \\
&\quad -\sigma I - \mu I & (4)\\
\frac{d_R}{d_t} &= \sigma I + \rho C^- - \mu R & (5)\\
\frac{d_{C^-}}{d_t} &= \theta C^+ - \rho C^- - \mu C^- & (6)\\
\frac{d_{C^+}}{d_t} &= W & \\
&\quad +\lambda(1-\psi)S_i + \lambda(1-\gamma)S_a + \lambda(1-\epsilon)S_c & \\
&\quad +\lambda(1-\psi)(1-\Delta_i)V_i + \lambda(1-\gamma)(1-\Delta_a)V_a + \lambda(1-\epsilon)(1-\Delta_c)V_c & \\
&\quad -\theta C^+ - \mu' C^+ & (7)\\
\frac{d_{Vi}}{d_t} &= Z' - cV_i - \lambda(1-\Delta_i)V_i - \mu V_i & (8)\\
\frac{d_{Vc}}{d_t} &= cV_i + \omega_c cS_i - aV_c - \lambda(1-\Delta_c)V_c - \mu V_c & (9)\\
\frac{d_{Va}}{d_t} &= aV_c + \omega_a aS_c - \lambda(1-\Delta_a)V_a - \mu V_a & (10)
\end{aligned}
$$

We take into consideration the susceptible proportion of the population (S_i, S_c, S_a, eq. 1-3), the chronic (C^+, C^-, eq. 6-7) and acute infections (I, eq. 4), the recovered and immune (R, eq. 5) and the vaccinated (V_i, V_c, V_a, eq. 8). Susceptible and vaccinated subgroups are divided into 3 main classes representing infants (S_i, <1 years of age), children (S_c, 1-6 years of age) and older individuals (S_a, >6 years of age).

Carriage and infection types

Carriers are represented by two chronic infection states depending on HBe-antigen status (designated C^+ for HBeAg-positive and C^- for HBeAg-negative), and I for acute infection. Individuals may acquire HBV at any of the age classes, developing chronic infection depending on age-associated risks: (1-ψ) for infants, (1-ε) for children, (1-γ) for older ages. We assume that the probability of developing chronic infections decreases with age, with ψ=0.15, ε=0.4, and γ=0.95 (1–3). When developing chronic infection, we assume that all individuals become HBeAg-positive but may lose this status and become HBeAg-negative at a rate θ (4). HBeAg-negative carriers may clear infection spontaneously at a rate ρ, entering the recovered class (R). Acute infections (I) are assumed to last 6 months (5) and are cleared at a rate σ, entering the recovered class (R).

Force of Infection

All carriers contribute to the force of infection (λ, eq. 11). It is assumed that chronic HBe-antigen positive infections (C^+) and acute infections (I) have a higher transmission rate ($\beta\beta_m$) than chronic HBe-antigen negative infections (C^-) (β) (6):

$$\lambda = \beta[C^- + \beta_m(C^+ + I)] \tag{11}$$

Births and Mortality

The population is assumed to be of constant size with equal births *b* (eq. 12) and deaths (μ, μ'). Mid-year population estimates from 2016 published by Statistics South Africa (7) were used to underpin assumptions about life expectancy. Due to HBV-associated mortality, the lifespan of chronic HBeAg-positive (C^+) individuals is taken to be lower (50 years) than the general lifespan (59 years (7)). In the absence of control, the total number of births (*b*) is divided into *Z* (eq. 13), *W* (eq. 14) and Z' (eq. 17) depending on the probability of vertical transmission (A_1, A_2) and proportion vaccinated at birth (ω_n). *W* is the proportion of babies born to infected mothers acquiring infection at birth or shortly after, and *Z* the proportion born susceptible.

$$b = \frac{\mu(S_a + S_c + S_i + I + R + V_i + V_c + V_a + C^-) + \mu' C^+}{S_a + S_c + S_i + I + R + V_i + V_c + V_a + C^- + C^+} \tag{12}$$

$$Z = b(1-\omega_n)(S_a + S_c + S_i + I + R + V_i + V_c + V_a) + bC^+(1-\omega_n)(1-A_1) + bC^-(1-\omega_n)(1-A_2) \tag{13}$$

$$W = bC^+A_1 + bC^-A_2 \tag{14}$$

Vertical Transmission

Vertical transmission takes place from mothers with chronic infections and is dependent on their HBe-antigen serostatus, with frequency of transmission α_1 for HBeAg-positive (C^+) and α_2 for HBeAg$^-$ (C^-). For interventions reducing vertical transmission, α_1 and α_2 are multiplied by (1-ζ), with ζ Є [0,1] being the impact of the intervention (eq. 15-16). For simplicity and lack of observations for appropriate parameterization, we assume that acute infections do not contribute to vertical transmission.

$$A_1 = \alpha_1(1-\zeta) \tag{15}$$

$$A_2 = \alpha_2(1-\zeta) \tag{16}$$

Routine vaccination

Routine vaccination is implemented under three general strategies: coverage of neonates (*Z'*, eq. 8, 17), coverage of 1-6 years old by vaccinating individuals leaving the susceptible <1 years old class (term $c\omega_c S_i$ in eq. 9), and coverage of 6+ years old by vaccinating individuals leaving the susceptible 1-6 years old class (term $a\omega_a S_c$ in eq. 10). In essence, we model vaccination occurring either at birth, or at particular ages (1 year, 6 years).

$$\begin{aligned} Z' &= b\omega_n(S_a + S_c + S_i + I + R + V_i + V_c + V_a) + \\ & \quad b\omega_n(1 - A_1)C^+ + b\omega_n(1 - A_2)C^- \end{aligned} \tag{17}$$

Catch-up vaccination

For simplicity, catch-up is modelled in a single event (time step t_{cu}), by moving a proportion of susceptible individuals into the age-corresponding vaccinated classes. In practice, this is an impulse event in the ODE system. Catch-up proportions are age-specific with parameters K_i for <1 years old, K_c for 1-6 years old, and K_a for 6+ years old.

$$K_i = \begin{cases} \kappa_i, & \text{if } t = t_{cu} \\ 0, & \text{otherwise} \end{cases} \tag{18}$$

$$K_c = \begin{cases} \kappa_c, & \text{if } t = t_{cu} \\ 0, & \text{otherwise} \end{cases} \tag{19}$$

$$K_a = \begin{cases} \kappa_a, & \text{if } t = t_{cu} \\ 0, & \text{otherwise} \end{cases} \tag{20}$$

Bayesian markov-chain Monte-Carlo fitting approach

In two independent steps, we fit certain ODE model outputs to empirically observed variables in the South African population, to set demographic and transmission backgrounds before simulating intervention strategies. We apply a Bayesian Markov-chain Monte-Carlo (MCMC) approach, developed and used by us in other modelling studies (8,9). The proposal distributions (*q*) of each parameter are defined as Gaussian (symmetric), effectively implementing a random walk Metropolis kernel. We define our acceptance probability *α* of a parameter set *Θ* given model ODE output *y* as:

$$\alpha = min\{1, \frac{\pi(y|\Theta^\star)p(\Theta^\star)q(\Theta^o|\Theta^\star)}{\pi(y|\Theta^o)p(\Theta^o)q(\Theta^\star|\Theta^o)}\} \tag{21}$$

where Θ^* and Θ^o are the proposed and current (accepted) parameter sets (respectively); $\pi(y \mid \Theta^*)$ and $\pi(y \mid \Theta^o)$ are the likelihoods of the ODE output representing the (observed)

variables by each parameter set Θ^* and Θ^o; $p(\Theta^o)$ and $p(\Theta^*)$ are the prior-related probabilities given each parameter set.

For simplicity and because all fitted variables are proportions, the likelihoods π were calculated as the product of conditional Gaussian probabilities *(Pr{...})*. The likelihood is the product the conditional probabilities of all variables. The likelihood can be formally expressed as:

$$\pi(y|\Theta) = \prod_{i=1}^{N} [Pr\{y_i = d_i\}] \qquad (22)$$

MCMC and model implementation

The mathematical ODE model and MCMC approach were developed in C/C++ (available as additional material which will be uploaded on manuscript acceptance). Visualisations were implemented in R.

Fitting demographic background

Before considering transmission and interventions, we first fitted the model to a demographic background. This is done with the above described fitting approach without transmission (i.e. at $t=0$, $I+C^{+}+C^{-} = 0$), using as target variables (Gaussian with standard deviation 1) the expected mean proportions of infants <1 years old ($S_i=0.022$), children 1-6 years old ($S_c=0.11$) and older ages ($S_a=0.868$) in the population of study (taken from Census 2011 (10)). We set the posteriors of the aging rates *a* and *c*, with median a=0.1337 (95% CI 0.1330 - 0.1343) and median c=0.7536 (95% CI 0.7369 - 0.7709). We set the values of *a* and *c* to the median values of the posteriors for all other model results (fitting transmission background and simulating interventions).

Fitting transmission background

After fitting demographic parameters and before considering interventions, we fitted the model to a transmission background. This is done using the above described fitting approach, with fixed aging rates *a* and *c*. The target variables are set to the percentage of the population that is HBsAg-positive (total carriers), percentage that are anti-HBc positive (*R*), and relative prevalences of chronic carriers HBeAg-positive (C^{+}) and HBeAg-negative (C^{-}) for the population of study. We used target Gaussian distributions (standard deviation 1) with mean 30% for anti-HBc, mean 8.3% for total carriers, mean proportion of 73% for HBeAg-negative and 23% for HBeAg-positive (6,11). In this step, the posteriors of the parameters β, ρ, α_1, α_2, θ and β_m are obtained.

Fitted parameters and priors for transmission setting

We fitted six parameters for the local transmission setting (β, ρ, α_1, α_2, θ and β_m). Gaussian informative priors are used for three parameters: frequency of vertical transmission α_1 for HBeAg-positive (C^+) with mean M=0.8 and standard deviation SD=0.05, the frequency of vertical transmission α_2 for HBeAg$^-$ (C^-) with M=0.25 and SD=0.05 (2,3,12,13), and the increased transmission factor for chronic HBe-antigen positive infections (C^+) and acute infections (*I*) β_m with M=10 and SD=2.5 (14–17). For β, ρ and θ uninformative, uniform priors are used with ranges of 0 to 30 for β and 0 to 1 for θ and ρ. In the main results we demonstrate that the posteriors for ρ and θ follow the scarce knowledge of these parameters.

Simulating deterministic interventions

After fitting demographics and transmission backgrounds, when simulating deterministic interventions, we fix *a*, *c*, β, ρ, α_1, α_2, θ and β_m to the obtained posterior medians. We vary combinations of the intervention parameters ω_n, ω_c, ω_a, (routine coverage for different ages), K_i, K_c, K_a (catch-up coverages) and ζ (reduction in vertical transmission). The transmission dynamics without interventions are run until the population reaches equilibrium, effectively reproducing the desired proportions as used in *Fitting transmission background*, at which point interventions are started and the model is tracked for 1000 years.

Simulating stochastic interventions

A stochastic version of the model presented in equations 1-10 was developed by introducing demographic stochasticity in state transitions. This followed a previously used strategy, in which multinomial distributions are used to sample the effective number of individuals transitioning between classes per time step (9,18,19). Multinomial distributions are generalized binomials – *Binomial (n,p)* - where *n* equals the number of individuals in each class and *p* the probability of the transition event (equal to the deterministic transition rate). Simulations followed the same approach as described for deterministic simulations (see above). However, for each combination of parameters defining the intervention, N=50 stochastic simulations are run by sampling N times the posteriors of the parameters obtained in *Fitting transmission background* (β, ρ, α_1, α_2, θ and β_m). This approach effectively takes into account demographic stochasticity and parameter (posterior) variation.

Fitting of cohort data on HIV serostatus and HBV vaccine-induced protection

We started with the assumptions that (i) protection is either constant or decays with age, (ii) vaccine efficacy reported elsewhere for infants is representative of protection levels in the population cohort of 1 year olds (infants), and (iii) HIV status may alter protection levels and decay of vaccine-mediated protection over time (20).

First, using a response threshold of ≥100 mIU/ml as a correlate of protection (20), we calculated the percentage of protected individuals in age 1, 2, 3, 4 and 5 years old, as available in the cohort data. Following assumption (i), we normalized the percentage of protected individuals in age by the percentage found for 1 year olds. Following assumption (ii) we multiplied this scaled variable ([0,1]) by an informed, literature-based baseline vaccine-induced protection (to infection) of 95% for HIV-negative infants and 75% for HIV-positive infants (see (20) for a recent literature review). The transformed protection cohort series are shown in red on Fig S1A,B. The obtained efficacy in the age group of 1 year olds is seen to be ~95% for HIV- and ~75% for HIV+, as expected.

We then used nonlinear weighted least-squares to fit the transformed protection cohort series (Fig S1AB) and projected protection in ages, with weights equal to the inverse of the (empirical) standard error for each age class (Fig S1C). The nonlinear model (Y~a*X^b) fitted the data closely (Fig S1A,B) for both HIV-positive and HIV-negative individuals (with resulting coefficients a=0.7842 b=-1.0477 for HIV-positive and a=0.95246 b=-0.05265 for HIV-negative). As reported elsewhere (20), projection of protection by age showed a significant difference depending on HIV serostatus, both in level of vaccine-mediated antibodies, and in decay of protection with age (Fig S1C).

Modelled HBV vaccine-induced protection in the context of HIV status

Given that the age classes in the dynamic model are discrete (<1, 1-6, 6+ years of age) and for simplicity, we parameterized protection according to the predicted (Gaussian) distributions at the mean age of each age class in the model (Fig S1D). That is, we used the predicted mean (M) and standard deviation (SD) at ages 0.5, 3.5, 32,5 years as proxies for protection at model age classes <1, 1-6, 6+ years of age, respectively. The resulting distributions (shown in Fig S1D-F) were: HIV-negative aged <1y with M=0.952 and SD=0.024, aged 1-6y with M=0.892 and SD=0.023, aged 6+y with M=0.796 and SD=0.074; HIV-positive aged <1y with M=0.784 and SD=0.148, aged 1-6y with M=0.217 and SD=0.070, aged 6+y with M=0.031 and SD=0.039. These estimations were in accordance

with previous studies and pooled ranges reported (20). Note that these values equate to protection at the individual level of each age class, such that, for example, HIV-negative aged <1y with M=0.952 equates to a mean of 95.2% vaccine-induced protection in that age class.

Vaccine-induced protection is modelled in the dynamic system using the term (1-Δx) in equations 4 and 7-10, where x relates to a specific age class. The term (1-Δx) therefore models a reduction in risk of infection, with Δx being the protection offered by the vaccine. Given that vaccine-induced protection is dependent on HIV status, Δx takes the following forms:

$$\Delta_i = P_i^+ \times v_i^+ + (1.0 - P_i^+) \times v_i^- \quad (25)$$
$$\Delta_c = P_c^+ \times v_c^+ + (1.0 - P_c^+) \times v_c^- \quad (26)$$
$$\Delta_a = P_a^+ \times v_a^+ + (1.0 - P_a^+) \times v_a^- \quad (27)$$

Where P_x^+ is the HIV prevalence at a certain age x, v_x^+ the vaccine-induced protection at a certain age x for HIV-positive individuals, and v_x^- the vaccine-induced protection at a certain age x for HIV-negative individuals (as determined in the approach detailed above). HIV prevalence levels used in the context of Kimberley were 1% for <1 years of age, 5% for 1-6 years of age, and 15% for >6 years of age (based on communications with clinicians in South Africa, (21)).

Obtained posteriors for informed and uninformed parameters

The posterior for the rate of seroconversion from HBeAg-positive to HBeAg-negative (θ) suggested slow progression (Fig S1B), with a median period of ~18.5 years (95% CI [14.3, 21.9]). We note here that although we used an uninformative (uniform) prior for θ, its posterior with median ~5.3% a year, here not accounting directly to age-specificity, is compatible with empirical estimations (22) of yearly rates of less than 2% for <3 years of age and 4-5% for older children (23), with ~90% of individuals acquiring HBV early in life remaining HBsAg-positive at the ages of 15-20 years (24).

Spontaneous clearance of chronic HBV infection (loss of HBsAg) (ρ) was estimated to be even slower (Fig S1B), close to 0.3% a year (95% CI [0.04, 0.84]), slightly lower than reported rates of 0.7-2.26% previously observed in the literature (25–27), although there remains a lack of data for the African subcontinent.

ADDITIONAL SUPPLEMENTARY FIGURES (FIG S1-S6)

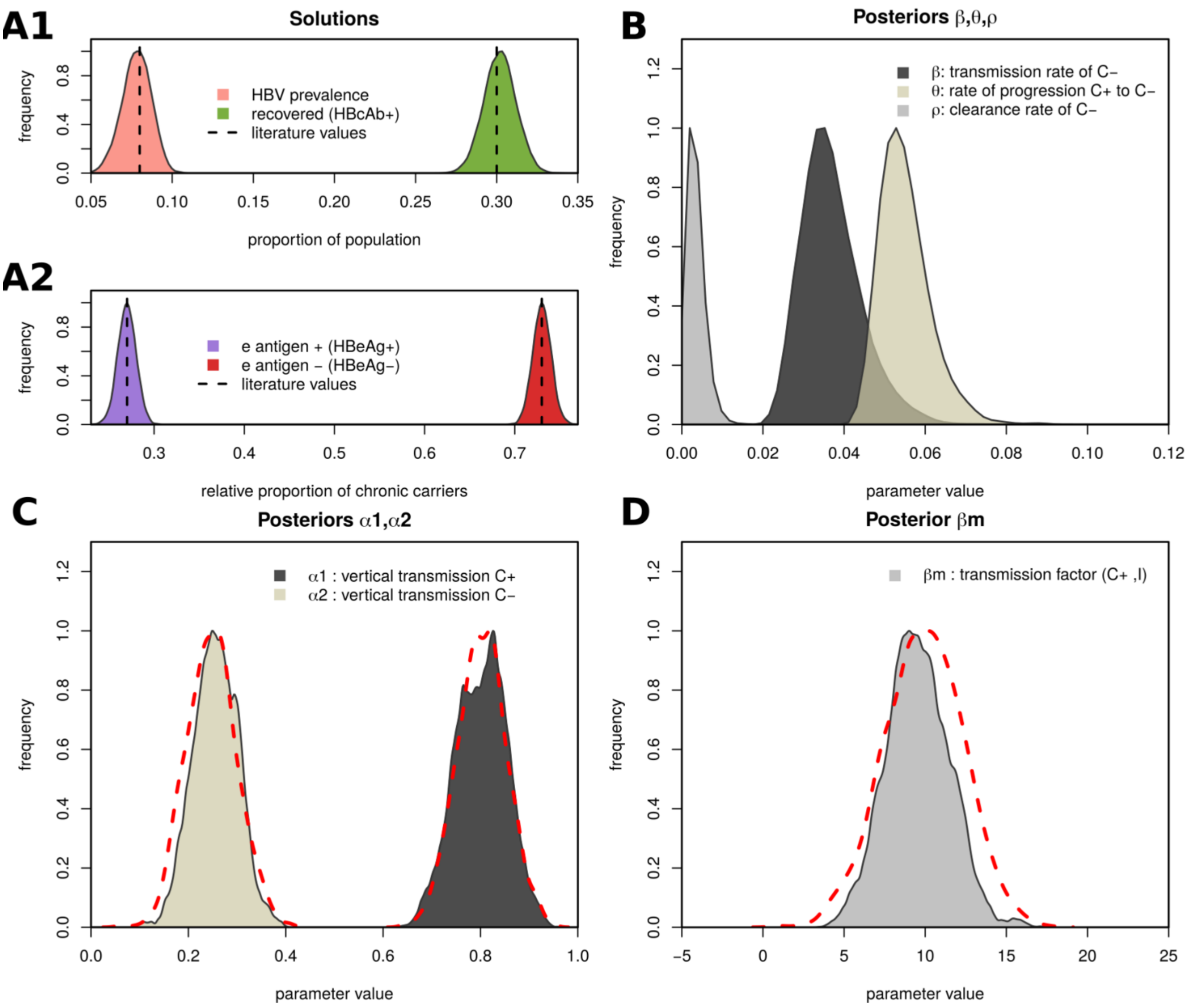

Additional Supplementary Figure 1 (Fig S1): Posteriors from the Bayesian Markov Chain Monte-carlo fitting to the Kimberley cohort data.

(A1-A2) Distributions of pre-intervention ODE model output at equilibrium for the fitted classes: (B1) carriers ($I+C^{+}+C^{-}$, HBsAg+, salmon) and recovered (*R*, HBcAg+, green); (B2) relative proportions of HBeAg^{+} (C^{+}, purple) and HBeAg^{-} (C^{-}, red) among chronic carriers ($C^{+}+C^{-}$). Distributions of target variables (fitted, B1, B2) are obtained by running the deterministic model with 10,000 samples of the posteriors shown in subplots C-E. Dashed vertical lines present the target fitted proportions based on the SA cohort and literature reports (see Methods Section). **(B-D)** Posterior distributions for the fitted parameters (1.5 million samples), with informative priors drawn with dashed red lines (1000 samples from distributions). Support results for the cohort data-driven approach related to HIV status and HBV vaccine-induced protection are in Additional Supplementary Fig S2.

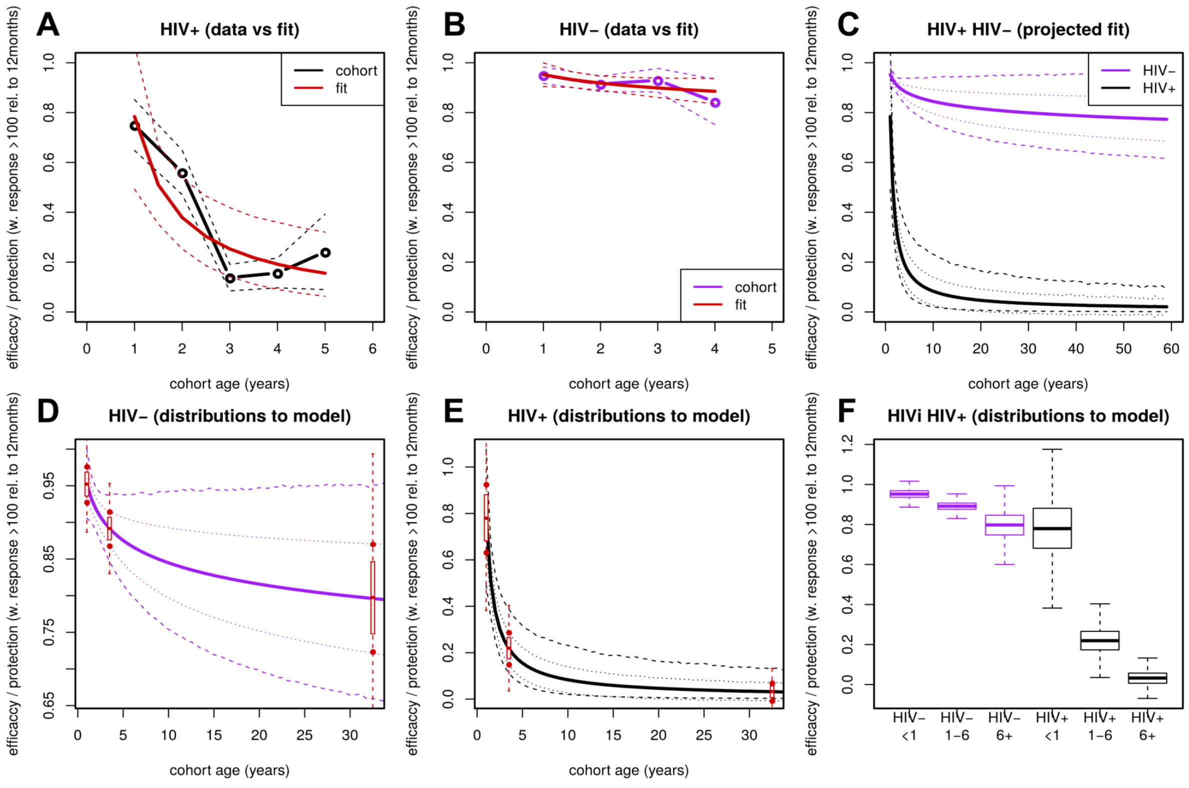

Additional Supplementary Figure 2 (Fig S2): Fitting HBV vaccine response according to HIV serostatus.

(A, B) Data on HBV vaccine response (see sections Waning of vaccine response with age, and Odds of developing an anti-HBs response) dependent on HIV serostatus. Data (points) and standard error (dashed) are shown in black for HIV+ (A) and purple for HIV- (B). Fit and 95% CI is shown in red. **(C)** Predicted HBV vaccine response dependent on HIV serostatus (HIV+ black, HIV- purple) across all ages. Dashed lines are the fitted 95% CI; dotted lines are the fitted standard deviation; solid bold lines are the fitted mean. **(D, E)** Boxplots in red show distributions obtained with 10,000 samples from a gaussian distribution with mean and standard deviation equal to the point prediction at mean ages of each age class in the dynamic model (0.5 years for class <1 years old, 3.5 years for class 1-6 years old, 32.5 years for age 1231 class 6+ years old). Distributions in subplot D are for HIV individuals and in subplot E are for HIV+ individuals. Red dots show the gaussian sampled standard deviation (which is seen approximating the fitted standard deviation). **(F)** Summary of the distributions found in subplots D and E according to HIV serostatus and later used in the dynamic model (HIV- in purple with <1y mean=0.952 std=0.024, 1-6y mean=0.892 std=0.023, 6+y mean=0.796 std=0.074; HIV+ in black with <1y mean=0.78 std=0.148, 1-6y mean=0.217 std=0.070, 6+y mean=0.031 std=0.039). (A-C) For fit details refer to methods section.

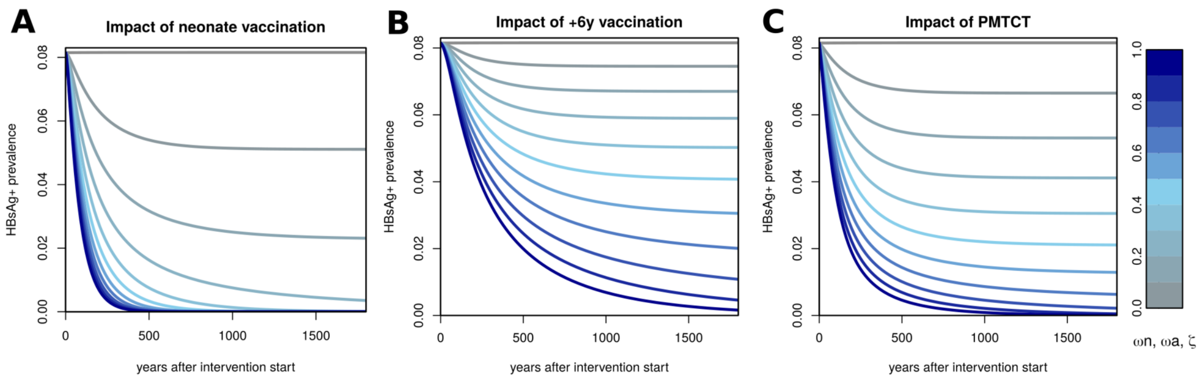

Additional Supplementary Figure 3 (Fig S3): Sensitivity of interventions with deterministic output.

Impact of **(A)** neonate vaccination (ω n), **(B)** vaccination at 6 years of age (ω a), and **(C)** PMTCT (ζ), on HBV prevalence (HBsAg) in time. The coverage / effort of simulated interventions quantified on the color scale to the right from 0 (no coverage / effort) to 1 (full coverage / effort). Impact is quantified by post-intervention reductions in HBV prevalence (HBsAg). Impact is highest for neonate vaccination, followed by PMTCT and lastly vaccination at 6 years of age for the same intervention effort. Simulations use the median parameter values of the posteriors shown in Support Figure 1. Results with stochastic simulations are presented in other figures of the main text and this additional file.

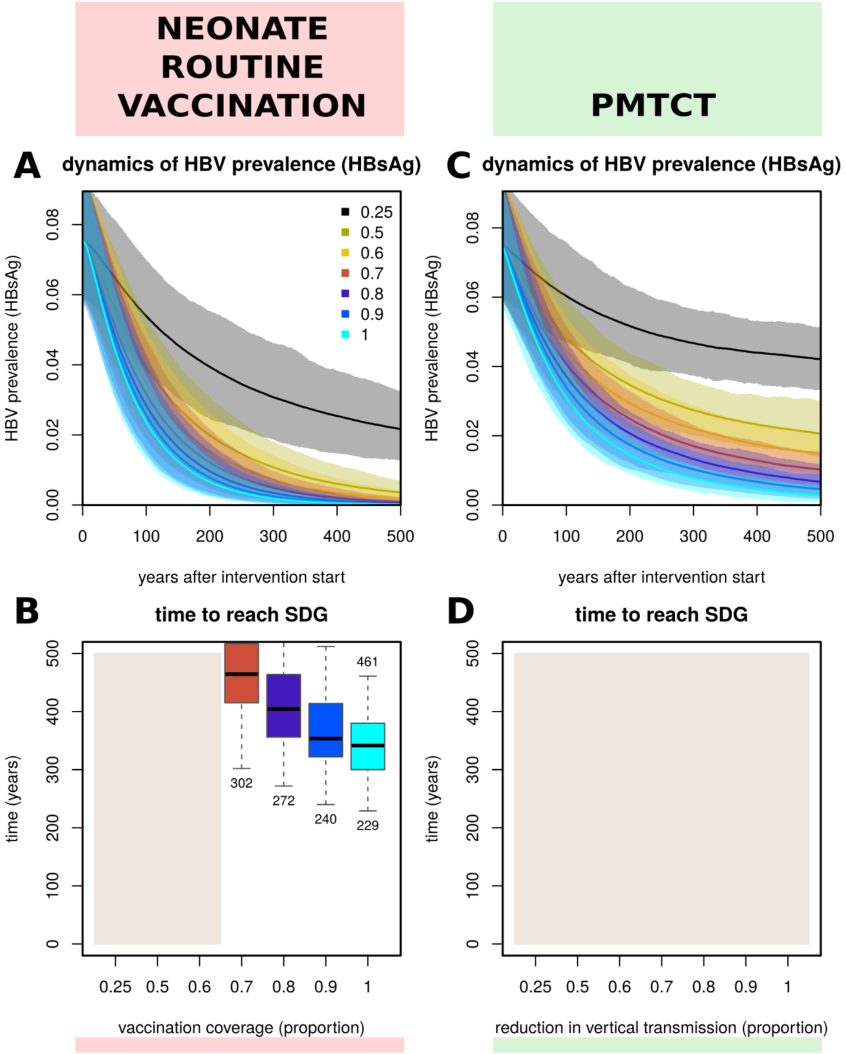

Additional Supplementary Figure 4 (Fig S4): Post-intervention stochastic impact on HBV prevalence (HBsAg), with time to reach sustainable development goals when using routine neonatal vaccination and PMTCT independently.

(A, B) Impact (reduction) on HBV prevalence (HBsAg) (A) and time to reach sustainable development goal (SDG) goal (B) for varying coverage of neonates. **(C, D)** Impact (reduction) on HBV prevalence (HBsAg) (C) and time to reach SDG (D) for varying PMTCT. (All subplots) Intervention coverage / effort varies from 0.25 to 1 (as colored and named in subplot A). **(A, C)** Lines are the mean and shaded areas are the standard deviation of model output when running 50 stochastic simulations per intervention (sampling the posteriors shown in Figure 1). **(B, D)** Beige areas mark interventions reaching SDGs after 500 years on average. Boxplots show the variation of the 50 stochastic simulations. Numbers above and below boxplots show the 2.5% lower and 97.5% upper limits of the solutions. The SDG is 1 in a 1000 individuals. **Compared to Figure 4 in the main text**: measuring impact with SDG on HBV incidence (HBsAg) (as opposed to HBV prevalence) results in more optimistic projections, i.e. shorter times to SDG (compare Figure 4 A2, C2 with this figure subplots B, D). PMTCT is unable to present solutions reaching the SDG for HBV prevalece (HBsAg) in 500 years (D).

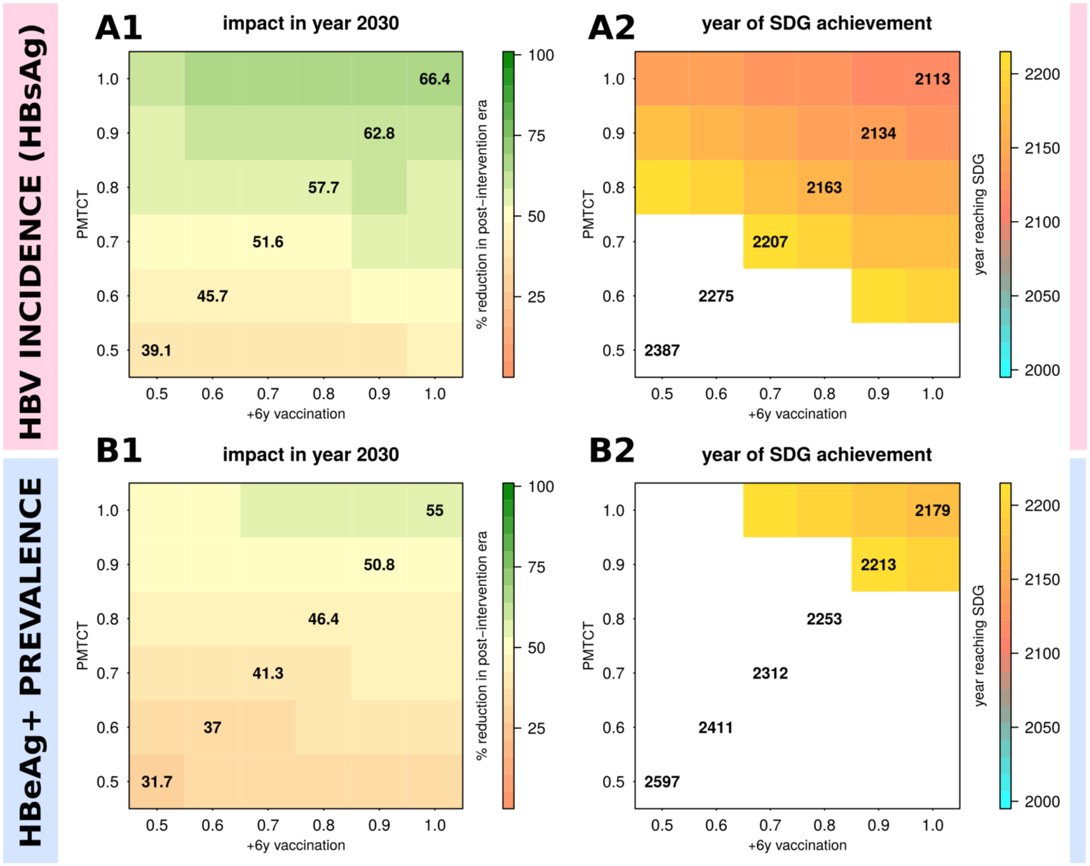

Additional Supplementary Figure 5 (Fig S5): Sensitivity of mean intervention impact on HBV incidence (HBsAg) and HBeAg+ prevalence, with estimated mean year to reach sustainable development goals for combinations of routine +6 years vaccination and PMTCT.

(A1-A2) Mean impact of interventions on HBV incidence (HBsAg) (A1) and mean time to reach sustainable development goals (SDGs) (A2). **(B1-B2)** Mean impact of interventions on HBeAg+ prevalence (B2) and mean time to reach SDG (B2). **(All subplots)** Impact is shown as percent reduction in incidence or prevalence compared to pre-intervention levels (e.g. 50 indicates a 50% reduction compared to last time step before intervention start). HBV incidence (HBsAg) SDG is set to a reduction of 90%. HBeAg+ prevalence SDG is set to 1 in a 1000 individuals. Mean results are obtained from 50 stochastic simulations per intervention combination (vaccination, PMTCT) with parameters sampled from the posteriors shown in Figure 1. Start of interventions in the stochastic simulations is in year 1995 to simulate an appropriate time scale to address impact by 2030. **Compared to Figure 5 main text:** the combination of PMTCT and routine vaccination of +6 years is highly suboptimal, with perfect routine coverage and PMTCT (top right cell, subplots A1, B1) achieving reductions of HBV incidence (HBsAg) and HBeAg+ prevalence by 2030 similar to half the vaccination coverage for neonates and half the PMTCT effort seen in Figure 5 (top right cell, subplots A1, B1), for example.

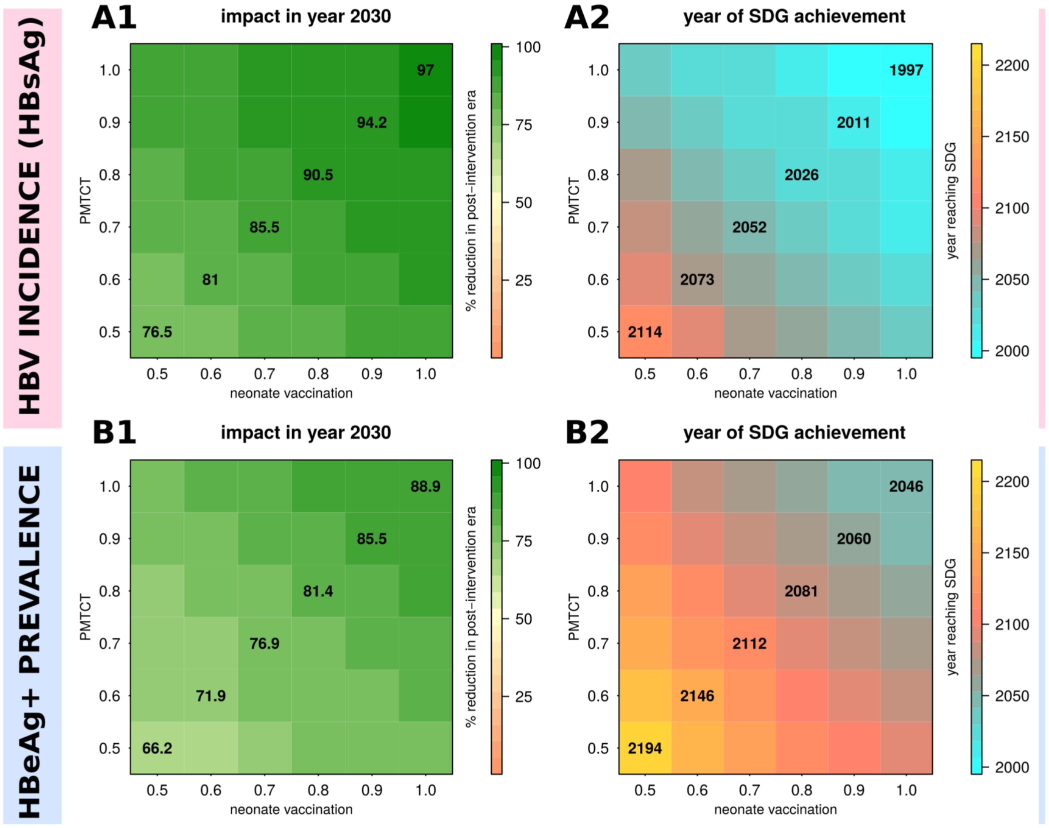

Additional Supplementary Figure 6 (Fig S6): Sensitivity of mean intervention impact on HBV incidence (HBsAg) and HBeAg+ prevalence, with estimated mean year to reach sustainable development goals for combinations of routine neonatal vaccination and PMTCT plus a complete catch-up campaign.

(A1-A2) Mean impact of interventions on HBV incidence (HBsAg) (A1) and mean time to reach sustainable development goals (SDGs) (A2). **(B1-B2)** Mean impact of interventions on HBeAg+ prevalence (B1) and mean time to reach SDG (B2). **(All subplots)** Impact is shown as percent reduction in incidence or prevalence compared to pre-intervention levels (e.g. 50 is 50% reduction compared to last time step before intervention start). HBV incidence (HBsAg) SDG is set to a reduction of 90%. HBeAg+ prevalence SDG is set to 1 in a 1000 individuals. Mean results are obtained from 50 stochastic simulations per intervention combination (vaccination, PMTCT) with parameters sampled from the posteriors shown in Figure 1. Start of interventions in the stochastic simulations is in year 1995 to simulate an appropriate time scale to address impact by 2030. Complete catch-up campaign is a one-off event with 100% coverage of all susceptible individuals in the population at the start of interventions. **Compared to Figure 5 main text**: adding 100% catch-up campaign to the interventions in Figure 5 is beneficial, for which the highest reductions of HBV incidence (HBsAg) and HBeAg+ prevalence by 2030 are achieved, as well as the shorter times to SDG. However, 100% catch-up is logistically and economically not feasible and the added benefits are small. For example, with complete neonatal coverage and PMTCT (top right cell, subplots A1, B1), the catch-up campaign would only add <5% in the mean reduction of HBV incidence (HBsAg) and HBeAg+ prevalence up to year 2030 (compare to top-right cells of subplots A1 and B1 in Figure 5).

REFERENCES

1. World Health Organization. Hepatitis B factsheet. 2017;http://www.who.int/mediacentre/factsheets/fs204/en.
2. Gentile I, Borgia G. Vertical transmission of hepatitis B virus: Challenges and solutions. International Journal of Women's Health. 2014;6:605–611.
3. Borgia G, Carleo MA, Gaeta GB, Gentile I. Hepatitis B in pregnancy. World Journal of Gastroenterology. 2012;18:4677–4683.
4. Fattovich G, Bortolotti F, Donato F. Natural history of chronic hepatitis B: Special emphasis on disease progression and prognostic factors. Journal of Hepatology. 2008;48:335–352.
5. Liang TJ. Hepatitis B: The virus and Disease. Hepatology. 2009;49:doi:10.1002/hep.22881.
6. Matthews PC, Beloukas A, Malik A, Carlson JM, Jooste P, Ogwu A, et al. Prevalence and characteristics of hepatitis B virus (HBV) coinfection among HIV-Positive women in South Africa and Botswana. PLoS ONE. 2015;10:e0134037.
7. Statistics South Africa. Mid-year population estimates. 2016;https://www.statssa.gov.za/publications/P0302/P030.
8. Faria NR, Da Costa AC, Lourenço J, Loureiro P, Lopes ME, Ribeiro R, et al. Genomic and epidemiological characterisation of a dengue virus outbreak among blood donors in Brazil. Scientific Reports. 2017;7:1–12.
9. Lourenço J, de Lima MM, Faria NR, Walker A, Kraemer MUG, Villabona-Arenas CJ, et al. Epidemiological and ecological determinants of Zika virus transmission in an urban setting. eLife. 2017;
10. Statistics South Africa. Census 2011 - Census in brief. 2012.
11. Schilsky ML. Hepatitis B "360." Transplantation Proceedings. 2013;45:982–985.
12. Degli Esposti S, Shah D. Hepatitis B and pregnancy: Challenges and Treatment. Gastroenterology Clinics of North America. 2011;40:355–72.
13. Piratvisuth T. Optimal management of HBV infection during pregnancy. Liver International. 2013;33:188–194.
14. Chu C-J, Hussain M, Lok ASF. Quantitative serum HBV DNA levels during different stages of chronic hepatitis B infection. Hepatology (Baltimore, Md.). 2002;36:1408–15.
15. Tong S, Kim KH, Chante C, Wands J, Li J. Hepatitis B Virus e Antigen Variants. Int J Med Sci. 2005;2:2–7.
16. Scaglioni PP, Melegari M, Wands JR. Biologic properties of hepatitis B viral genomes with mutations in the precore promoter and precore open reading frame. Virology. 1997;233:374–381.
17. Hasegawa K, Huang J, Rogers S a, Blum HE, Liang TJ. Enhanced replication of a hepatitis B virus mutant associated with an epidemic of fulminant hepatitis. Journal of virology. 1994;68:1651–9.
18. Lampoudi S, Gillespie DT, Petzold LR. The multinomial simulation algorithm for discrete stochastic simulation of reaction-diffusion systems. Journal of Chemical

Physics. 2009;130:1–16.

19. Lourenço J, Recker M. The 2012 Madeira Dengue Outbreak: Epidemiological Determinants and Future Epidemic Potential. PLoS Neglected Tropical Diseases. 2014;8.

20. Mena G, García-Basteiro AL, Bayas JM. Hepatitis B and A vaccination in HIV-infected adults: A review. Human vaccines & immunotherapeutics. 2015;11:2582–98.

21. Li W, Urban S. Entry of hepatitis B and hepatitis D virus into hepatocytes: Basic insights and clinical implications. Journal of Hepatology. 2016;64:S32–S40.

22. Liaw YF. HBeAg seroconversion as an important end point in the treatment of chronic hepatitis B. Hepatology International. 2009;3:425–433.

23. Chang M, Hsu H, Hsu H, Ni Y, Chen D. The Significance of Spontaneous Hepatitis B e Antigen Seroconversion in Childhood : With Special Emphasis on the Clearance of Hepatitis B e Antigen Before 3 Years of Age. Hepatology. 1995;5:1387–92.

24. Chu CM, Sheen IS, Lin SM, Liaw YF. Sex difference in chronic hepatitis B virus infection: studies of serum HBeAg and alanine aminotransferase levels in 10,431 asymptomatic Chinese HBsAg carriers. Clinical infectious diseases. 1993;16:709–713.

25. Ferreira SC, Chacha SG, Souza FF, Teixeira AC, Santana RC, Villanova MG, et al. Factors associated with spontaneous HBsAg clearance in chronic hepatitis B patients followed at a university hospital. Ann Hepatol. 2014;13:762–770.

26. Liu J, Yang HI, Lee MH, Lu SN, Jen CL, Wang LY, et al. Incidence and determinants of spontaneous hepatitis B surface antigen seroclearance: A community-based follow-up study. Gastroenterology. 2010;139:474–482.

27. Chu CM, Liaw YF. HBsAg seroclearance in asymptomatic carriers of high endemic areas: Appreciably high rates during a long-term follow-up. Hepatology. 2007;45:1187–1192.
